# The Complosome: An Emerging Intracellular Complement Network in Cancer Development and Therapy

**DOI:** 10.3390/ijms27094111

**Published:** 2026-05-04

**Authors:** Łukasz Zadroga, Filip Lewandowski, Dominika Bębnowska, Adam Majchrzak, Alina Czyż, Paulina Niedźwiedzka-Rystwej

**Affiliations:** 1 Department of General, Dental and Interventional Radiology, Pomeranian Medical University in Szczecin, 70-111 Szczecin, Poland; zadroga09@gmail.com; 2 Institute of Biology, University of Szczecin, 71-412 Szczecin, Poland; filip.lewandowski@usz.edu.pl (F.L.); dominika.bebnowska@usz.edu.pl (D.B.); 3 Center for Experimental Immunology and Immunobiology in Infectious Diseases and Cancer, University of Szczecin, 71-412 Szczecin, Poland; a.czyz@opoczta.pl; 4 Doctoral School, University of Szczecin, 71-412 Szczecin, Poland; 5 Department of Infectious, Tropical Diseases and Acquired Immunodeficiency, Pomeranian Medical University in Szczecin, 71-455 Szczecin, Poland; adammajchrzak@proton.me; 6 Regional Centre for Digital Medicine, Pomeranian Medical University in Szczecin, 70-204 Szczecin, Poland

**Keywords:** complosome, cancer, complement, tumor development, immune evasion

## Abstract

The complement system is traditionally recognized as a major effector of innate immunity, essential for pathogen clearance, inflammation and the maintenance of tissue homeostasis. In recent years, however, its role in cancer has been substantially redefined. Beyond its canonical extracellular activity, complement has emerged as a multifaceted regulator of tumor biology, acting not only within the tumor microenvironment but also intracellularly through the recently described intracellular complement system (complosome). While extracellular complement primarily shapes immune responses and the tumor microenvironment, the complosome directly regulates fundamental cellular processes, including metabolism, proliferation, autophagy, stress responses and cell survival. In this review, we discuss current evidence on the canonical and non-canonical roles of complement in cancer. Importantly, complement signaling exhibits a strong context-dependent duality, exerting either tumor-promoting or tumor-restraining effects depending on the tumor type, disease stage, cellular source, and localization. Taken together, the available evidence indicates that the complosome is not merely an extension of classical complement biology, but a distinct and biologically significant signaling network that rewrites our understanding of complement in cancer. Its growing relevance in tumor development and therapy resistance positions it as a promising target for future mechanistic studies and innovative therapeutic interventions.

## 1. Introduction

Malignant tumors remain one of the diseases with the highest mortality rates in the world, and their incidence continues to rise steadily due to an aging population and lifestyle changes [1]. According to the latest calculations, there were almost 4.5 million new cases of cancer in Europe in 2022, with an age-standardized incidence and mortality rate (ASR) of 280 per 100,000 people, and a cumulative risk of developing cancer by the age of 75 of 27.9% [2]. World Health Organization (WHO) projections indicate that in 2050, the number of new cancer cases will exceed 35 million, representing a 77% increase compared to the estimated 20 million cases in 2022 [3]. Despite enormous advances in diagnosis and treatment, cancer remains a serious health and social challenge. Moreover, cancer generates a significant economic burden—both for healthcare systems and for patients. According to estimates, more than half of patients face substantial increases in healthcare costs, leading to significant deterioration in quality of life [4].

The complement system is a key component of innate immunity, acting as the first line of defense in the organism, responsible for recognizing and eliminating pathogens, as well as removing damaged host cells [5]. The complement system consists of numerous plasma and cell membrane proteins that interact in a cascade of enzymatic reactions activated by three main pathways: the classical (CP), lectin (LP), and alternative (AP) pathways, which are part of the canonical function of complement. The common effect of the activation of these three pathways is the generation of key molecules: opsonins and anaphylatoxins, which support phagocytosis and have strong pro-inflammatory and chemotactic properties, as well as the membrane attack complex (MAC), which leads to cell lysis [6]. The non-canonical functions of complement relate to its involvement in cell differentiation, chemotaxis, adhesion, and migration. In addition, the system also participates in the remodeling of the extracellular matrix and the removal of apoptotic cells [7]. Avoiding the immune response is a well-known phenomenon in the context of cancer. In recent years, there has been growing evidence that complement components are targeted for manipulation in the tumor microenvironment (TME) [8]. Cancer cells can use the complement system to remodel the TME, promote tumor growth and metastasis, and suppress the immune response, including the antitumor activity of T cells [9]. In addition, the discovery of the intracellular complement system (complosome), an intracellular complement system activated independently of circulating components, has opened a new chapter in research into its role in regulating cell metabolism, proliferation, and survival [10].

The complement system participates in tumor development not only through its action in the tumor microenvironment, but also intracellularly as the complosome, playing a dual, complex role in tumor progression and immune response regulation.

## 2. Tumor Microenvironment (TME) as a Complex Ecosystem

The tumor microenvironment (TME) consists of cellular and extracellular components that together form a complex ecosystem influencing tumor development [11]. The cellular components of the TME include cancer cells as well as a heterogeneous population of non-malignant cells [12,13]. These comprise numerous leukocytes, including various T-cell subpopulations (cytotoxic, helper and regulatory T-cells, Treg) [14], as well as B lymphocytes [15], NK cells [16], macrophages (including tumor-associated macrophages, TAMs) [17], neutrophils [18], dendritic cells [19] and myeloid-derived suppressor cells (MDSCs) [20]. Stromal cells also play a crucial role, including fibroblasts (particularly activated cancer-associated fibroblasts, CAFs) [21] and cells of the blood and lymphatic vessels (endothelial cells and pericytes) [22,23]. The extracellular components of the TME include the extracellular matrix (ECM), composed mainly of collagens and other structural proteins (e.g., fibronectin, laminins and proteoglycans), as well as soluble mediators (cytokines, chemokines and growth factors) and an abnormal network of blood and lymphatic vessels [24]. The TME is increasingly understood as a complement-regulated niche in which tumor cells, infiltrating immune cells and stromal cells use not only extracellular complement but also the intracellular complement system or the complosome, to control cell fate [25]. The complosome comprises complement proteins, receptors, and regulators that are synthesized, retained, processed and activated within cells, where they regulate metabolism, survival, inflammasome activity, autophagy and immune signaling [26]. Accordingly, the relevance of the TME to cancer progression should not be discussed only as a matter of cellular composition, but rather as the outcome of complement-dependent programs operating within malignant, immune and stromal compartments. In this sense, the TME is best viewed as a spatially organized network in which extracellular complement and cell-intrinsic complement signaling cooperate to shape tumor growth, immune escape and therapy response [27]. Within tumor cells, intracellular complement activation directly promotes malignant fitness [28]. In colorectal cancer, intracellular C5 is cleaved by cathepsin D, generating C5a that signals through intracellular C5aR1 to stabilize β-catenin and thereby promote tumorigenesis [29,30]. In renal cancer, tumor-associated classical pathway activity, including C4 activation products, correlates with poor prognosis [31], while C1s exerts non-canonical functions that support tumor cell proliferation and dampen T-cell activation, indicating that classical pathway complement components can be coopted for tumor cell-autonomous functions beyond canonical extracellular complement activation [28]. The tumor intrinsic complosome also interfaces with metastasis, cell stress and non-canonical complement regulators. A key example is the sphingolipid pathway: oncogenic sphingosine-1-phosphate signaling induces intracellular C3 activation, promotes the formation of the C3b-α′2 fragment, enables PPIL1 engagement and activates the NLRP3 inflammasome, collectively enhancing cancer cell migration, invasion and metastatic lung colonization [32]. In parallel, intracellular factor H in clear cell renal cancer and tumor-promoting fibroblasts promotes proliferation, cell cycle progression, cytoskeletal remodeling and resistance to growth suppression through mechanisms linked to Rb/E2F, p53, and actin-capping complexes rather than to its canonical extracellular complement regulatory role [33]. Emerging data also suggest that factor B family signaling can participate in intracellular complement metabolic circuits, but here the strongest recent mechanistic support currently comes from fibroblast and inflammatory models rather than from fully established tumor cell intrinsic cancer models [32,34]. A second major role of the complosome in the TME is the regulation of immune cell activation, differentiation and persistence. Tissue entry itself induces intrinsic C3 expression in immune cells, including T cells and monocytes, showing that intracellular complement activation is a physiological feature of tissue-resident immunity rather than a passive consequence of serum complement exposure [35]. In CD4+ T cells, cell-autonomous C5 activation and intracellular C5aR1 signaling promote NLRP3-dependent IL-1β secretion and support Th1 effector function, whereas a distinct C5aR2-prostacyclin-IL-1R2 axis mediates Th1 contraction [36]. These observations are highly relevant for tumor biology because they demonstrate that intracellular complement in T cells does not merely amplify inflammation, but controls the magnitude, quality, and duration of antitumor immune responses through metabolic and inflammatory checkpoints. In tumors, however, complement-linked immune signaling is frequently redirected toward immunosuppression [37]. Intracellular activation of tumor cell-derived C3 can generate C3a that acts on tumor-associated macrophages through the C3a-C3aR-PI3Kγ axis, suppressing CD8+ T-cell responses and contributing to resistance to PD-L1 blockade [38]. Similarly, C5a/C5aR1 signaling in ovarian cancer skews TAMs toward an immunosuppressive phenotype, whereas genetic or pharmacologic C5aR inhibition restores macrophage anti-tumor activity, enhances CXCL9-dependent CD8+ T-cell function, and improves the efficacy of immune checkpoint blockade [39]. Recent immunotherapy-focused reviews also continue to identify C5a as a recruiter of MDSCs and Treg cells, placing the complosome within a broader complement-driven suppressive circuit that weakens effective cytotoxic lymphocyte activity in solid tumors [40,41]. Taken together, these findings demonstrate that intracellular complement activation integrates metabolic regulation, immune-cell polarization and the suppression of cytotoxic responses across multiple immune cell populations within the TME. A structured overview of complosome-driven mechanisms and their functional consequences is presented in Table 1.

The complosome further shapes tumor–immune–stromal communication through non-canonical complement crosstalk. Tumor cells can cooperate with macrophages to assemble a locally active classical-pathway circuit, in which TAM-derived C1q and tumor cell-derived C1r, C1s, C4, and C3 promote a pro-tumor inflammatory program associated with poor prognosis and an immune checkpoint-rich microenvironment [41,49]. In thyroid cancer, a recent study identified tumor-derived CFI as a macrophage-polarizing signal that promotes M2-like macrophage differentiation while lowering C5a [45]. In glioblastoma, extracellular vesicles carrying tumor-derived C3 promote metastatic spread by polarizing TAMs and recruiting PMN-MDSCs [50]. Although not every one of these pathways satisfies the strictest intracellular definition of the complosome, together they demonstrate that tumor-intrinsic complement production and intracellular-complement-regulated states can propagate across the TME to reinforce immune evasion and metastatic niche formation [50,51]. Stromal biology should also be included explicitly, because the emerging evidence no longer supports limiting the complosome to tumor cells and leukocytes. Beyond oncology, fibroblast-focused mechanistic studies now show that intracellular C3, CFB, and CFD can drive metabolic and proinflammatory reprogramming, increasing glycolysis, TCA-cycle activity, fatty-acid metabolism, ATP-linked respiration and inflammatory mediator production through intracellular complement signaling [52,53]. These data do not yet define the complosome in CAFs equally well across all tumor types, but they strongly support the view that stromal cells can harbor complement-intrinsic circuits capable of remodeling tumor ecology [53]. A recurring theme across all of these studies is that intracellular complement acts as a metabolic-inflammatory hub. The complosome intersects with glycolysis, oxidative phosphorylation, mitochondrial activity, inflammasome assembly, autophagy, apoptosis and cellular stress adaptation [33,54]. This is especially clear under hypoxia: low oxygen induces C5aR1 expression in a UPR-dependent manner, increases the endocytic relocalization of C5aR1 to intracellular compartments and enables cancer cell survival by regulating autophagy and apoptosis [33,55]. For this reason, the complosome should be regarded as functionally distinct from extracellular complement and therefore as a separate therapeutic target. The translational relevance of this idea is already supported by preclinical studies showing that C5aR1 blockade reprograms macrophages and enhances checkpoint blockade [56], as well as by early clinical targeting of factor H with GT103 in refractory non-small-cell lung cancer [57]. Collectively, the available evidence indicates that the complosome acts across tumor cells, immune cells and stromal cells to promote proliferation, survival, immune evasion, metastatic dissemination and therapy resistance, making it a plausible next-generation target in cancer therapy [41,50,58]. Importantly, immune cells within the TME do not respond uniformly to complosome activity but instead undergo distinct, context-dependent functional adaptations involving metabolic rewiring, polarization, and intercellular signaling. These response patterns across major immune cell subsets are summarized in Table 2.

## 3. Complement Activation: The Classical, the Lectin, and the Alternative Pathway

**Figure 1:**
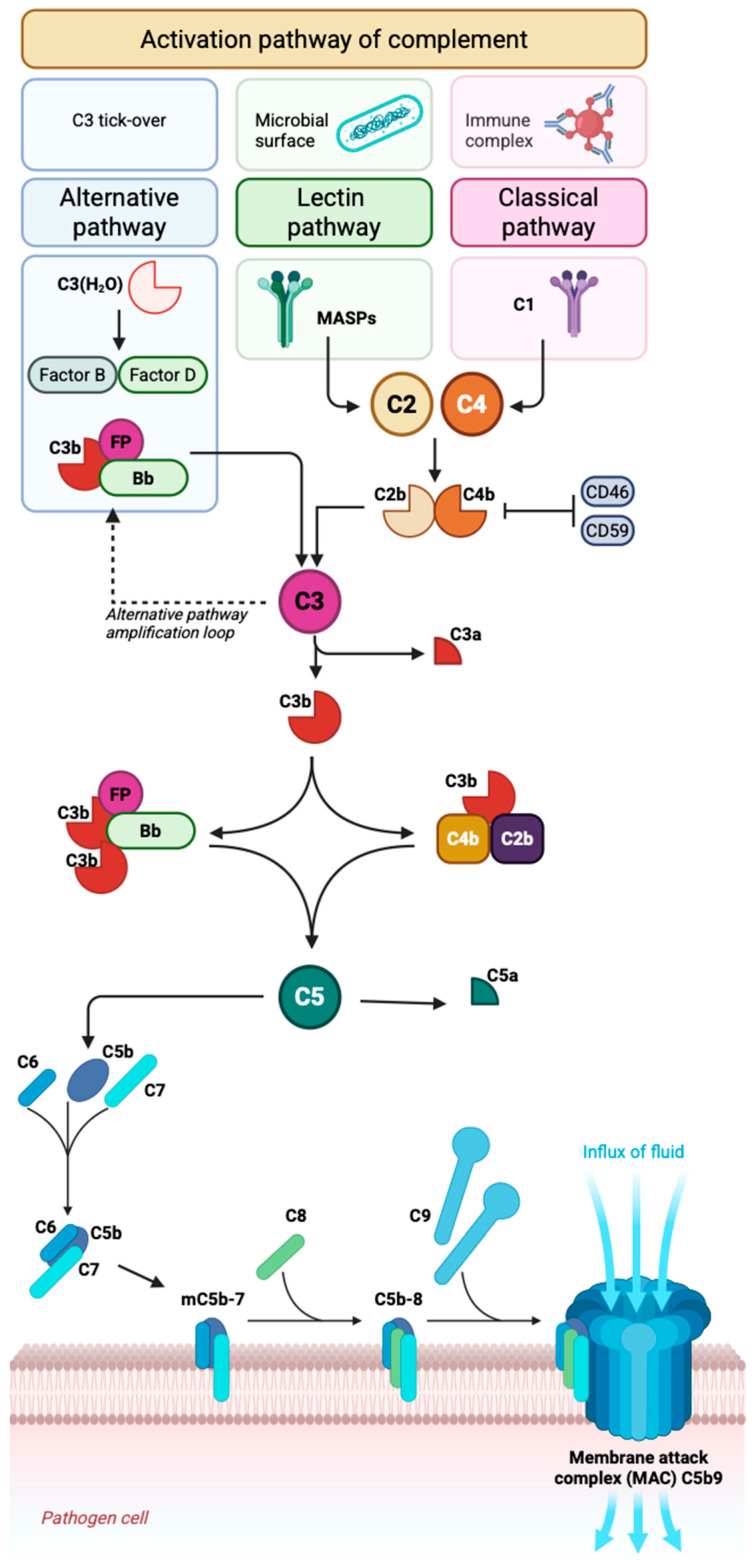
Overview of classical, lectin, and alternative complement activation pathways leading to C3/C5 convertases and membrane attack complex (MAC) assembly. The classical pathway is initiated by C1 binding to antigen–antibody immune complexes, while the lectin pathway is triggered by recognition of microbial carbohydrates by lectins and activation of MASPs. The alternative pathway is constitutively activated via spontaneous C3 “tick-over” and amplified by Factor B, Factor D, and properdin. All pathways converge at C3 cleavage, generating C3a and C3b, and subsequently form C5 convertases, leading to cleavage of C5 into C5a and C5b. C5b initiates assembly of the membrane attack complex (MAC; C5b–9), resulting in pore formation and target-cell lysis. Complement activity is regulated by surface inhibitors, including CD46 and CD59, with CD59 preventing MAC formation.

There are three known activation pathways in the complement system: the classical pathway (CP) [64], the lectin pathway (LP) [6] and the alternative pathway (AP) [64] (Figure 1).

CP activation occurs through antibody binding to the C1q–C1r/C1s complex [25]. As a result, serine proteases are activated, which lead to the cleavage of C4 and C2, generating the C3 convertase (C4b2a). C3 convertase cleaves C3 into C3a and C3b. Then, C3b binds to the C4b2a complex to form the C5 convertase (C4b2a3b). C5 convertase triggers the formation of the membrane attack complex (MAC), which inserts into the bacterial membrane, forming pores that lead to its lysis [6,64].

The formation of MAC is primarily regulated by the complement regulatory proteins, including: CD46, CD55, CD59, factor I and factor H [65,66,67]. CD59, by binding with the C9 and C8 components, blocks MAC polymerization directly [68,69]. CD55 destabilizes C3/C5 convertases through its binding with C3b and C4b, thereby indirectly preventing MAC formation [65]. CD46 is a cofactor of factor-I-mediated enzymatic cleavage of activated C3b/C4b in the presence of factor H [67].

The lectin pathway is similar to the classical pathway and is activated by MBL (mannose-binding lectin), ficolins, and collectins, thereby activating MASP-1 or MASP-2 (mannan-binding lectin-associated serine proteases-1 or -2) [6,25,64,70]. MASP-2 cleaves C4, generating C4a and C4b [25], whereas MASP-1 cleaves C2, generating C2a and C2 [6]. MASP-2 and MASP-1 together lead to the formation of the C4bC2a complex [25,70]. Additionally, MASP-1 can directly cleave C3, bypassing the C4bC2a complex. Activation of the CP and LP leads to the formation of the key C3 convertase, C2aC4b [25,64,70].

The alternative pathway is not a cascade trail initiated by a specific factor, but rather a self-amplifying loop driven by the C3 convertase [71]. The thioester bond within C3 is highly reactive and undergoes spontaneous hydrolysis, leading to the formation of C3(H_2_O), a molecule structurally analogous/similar to C3b [64]. C3(H_2_O) binds factor B, which, after proteolytic cleavage, produces a soluble C3 convertase capable of generating additional C3b molecules [72]. Complement regulators present on healthy cells maintain control over the spontaneous hydrolysis of C3. A lack of C3b inactivation results in the amplification of the alternative pathway [64,71,72].

C3b becomes activated upon binding to factor B. Factor D subsequently cleaves factor B into the Ba and Bb fragments [73]. The Bb fragment remains associated with C3b, forming the alternative pathway C3 convertase, C3bBb. In the presence of factors B and D, C3b can generate new C3 convertase molecules, functioning as an ‘amplification loop.’ The alternative pathway bypasses components C1, C2, and C4 [6,25,64,73].

## 4. The Complosome: An Emerging Intracellular Immune Network Shaping Cellular Homeostasis and Tumor Fate

In recent years, research findings have indicated an important role of complement acting inside cells; however, the mechanisms underlying its canonical and non-canonical functions remain largely unknown [25,63]. Intracellular complement activity has been detected in a wide range of cell types examined so far, including fibroblasts, hepatocytes, epithelial cells of the lungs, intestines, and retina, as well as renal endothelial cells and pancreatic β-cells, highlighting its broad physiological significance [74].

Key components of the complosome, such as C3 and C5 and their receptors [25,27,46,75,76], have been detected in the cytoplasm [27], lysosomes [46], the endoplasmic reticulum [75], the outer mitochondrial membrane [76], and even the cell nucleus [25]. These diverse locations enable the complosome to perform functions distinct from those known in the classical complement biology. It is involved in crucial cellular processes such as metabolism, autophagy, and the regulation of gene expression [25,27,46,75,76].

Consequently, the complosome supports the maintenance of cellular balance by modulating cellular responses to both infectious and non-infectious stressors and by facilitating the return to homeostasis [25,27,63,74]. Intracellular C3aR and C5aR1 receptors present in mitochondria, lysosomes and endosomes, exert their effects through the activation of signaling pathways similar to those observed for classical surface receptors [46,76]. However, most complosome functions are non-canonical, resulting/arising from their unique ability to interact with selected cellular components isolated from extracellular complement activity [27]. Intracellular C3 protects airway epithelial cells from oxidative stress and a lack of nutrients, and in retinal epithelial cells, the activation of mitochondrial C3aR reduces mitochondrial respiration, thereby limiting oxidative stress. Additionally, C3 is associated with the regulation of autophagy in response to both bacterial and non-infectious stress, supporting the maintenance of cellular homeostasis [74].

The complosome is integral to the function of cytotoxic T lymphocytes (CTLs) [25], neutrophils [27], macrophages [6], and monocytes [74]. In neutrophils, it regulates cellular biomechanics through cytoskeletal reorganization [27], whereas in macrophages, it slows phagosome–lysosome fusion after antigen opsonization by C3b, controlling the rate of antigen processing and T-cell responses [6]. Mitochondrial C5aR1 signaling supports the structure, morphology, and metabolism of resting monocytes, and epithelial cells respond to non-infectious stressors by modulating local complement activity, for example, through FHR-3 or mitochondrial C3aR [27].

**Figure 2:**
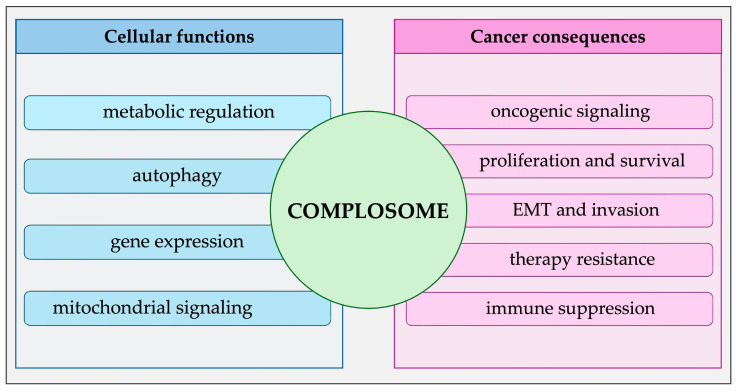
Summary of the cellular functions and cancer consequences of complosome. The diagram illustrates the dual role of the complosome as a central regulator linking fundamental cellular processes with oncogenic outcomes. The left side shows key physiological functions modulated by the complosome and the right side summarizes consequences of complosome dysregulation in cancer.

Complement components play a significant role in cancer, participating in angiogenesis, invasion, metastasis, modulation of immune responses, metabolic reprogramming, remodeling of the tumor microenvironment, uncontrolled proliferation, and therapy resistance (Figure 2) [6,25]. Although classical complement activation pathways are involved in these processes, the role of complosome components in cancer often occurs independently of classical activation [25,27,77]. Intracellular and locally produced complement proteins, such as C1q, C1r, C1s, C3, and C5, exert mainly pro-tumorigenic effects independently of complement activation [6,25,43,63,64,70]. The C3a–C3aR [6] and C5a–C5aR [43] interactions activate PI3K/AKT/mTOR pathways, which promote protein synthesis, proliferation, survival, and the motility of cancer cells. C3 initiates JAK2/STAT3 and ERK signaling pathways, metabolically reprogramming the cell to favor tumor progression [6,25,70]. Tumor fate depends on dynamic interactions between complement components, immune cells, and the tumor microenvironment (TME) [6,25]. The functions of complement system components can be pro- or anti-tumorigenic, depending on the tumor type, stage, and the immunological characteristics of the TME [25,77].

## 5. Complosome and Non-Canonical Complement Signaling in Tumor Progression and Immune Evasion

The complement system influences tumor cell homeostasis and immune evasion in ways that extend beyond its classical roles of lysis and opsonization. As summarized in Table 3 and Table 4, the complement system operates both intracellularly via the complosome and extracellularly, in a non-classical way through individual components and regulators, exhibiting both pro- and anti-tumor effects.

### 5.1. C1q/C1qR

In malignant pleural mesothelioma (MPM), C1q linked to hyaluronic acid (HA) serves as an essential regulatory element. By upregulating hyaluronidase 2 (HYAL2) expression, C1q accelerates HA catabolism into low-molecular-weight HA (LMW-HA) polymer fragments. In the TME of mesothelioma, LMW-HA fragments served as pro-inflammatory and pro-tumorigenic mediators [78]. Beyond that, C1q/C1qR axis mediates the mitochondrial-driven oxidative metabolism of respiratory chain enzymes. During chronic inflammation, the selective cleavage of the gC1qR by caspase-1 occurred. This hinders gC1qR transport into mitochondria, consequently redirecting cancer cell metabolism towards aerobic glycolysis and promoting a hypoxic environment. These anaerobic conditions within the colorectal cancer TME drive tumorigenic progression, increasing cancer cell proliferation [79].

### 5.2. C1r/C1s

In cutaneous squamous cell carcinoma (cSCC) silencing of the C1r and C1s genes induces apoptosis and reduces the proliferation of cSCC. C1s is activated extracellularly by the enzymatic cleavage of the C1s proenzyme. Active C1s intensifies tumor cell proliferation and invasiveness via the Akt and ERK1/2 pathways [80]. Additionally in cSCC, elevated C1r levels induce expression of metalloproteinases MMP1, MMP13, MMP10, and MMP12, which enhance collagen degradation, increasing invasiveness of tumor cells [81]. In SCC cells anti-C1s antibodies are present both intracellularly and extracellularly, indicating the C1s presence within the cancer cells. Antibody-mediated C1s blockage promotes apoptosis and inhibits the growth of SCC cell lines. Protein level analysis of pathways related to SCC cells growth showed that C1s blockage inhibits the ERK1/2 and Akt signaling pathways, thereby suppressing SCC growth [82]. C1s plasma levels negatively impact prognosis in early stages (I and II), but not in the advanced stages (III and IV) of renal cell cancer (RCC). The number of C1s+ cells correlates with increased CD8+ T-cell infiltration and PD-1 expression. Following C1s silencing, a higher count of CD38+ CD4+ and CD8+ T cells was measured in the presence of tumor cells, implying a tumor-intrinsic effect of C1s [83].

### 5.3. Intracellular C3/C3aR

C3AR is predominantly expressed in the intracellular region of cancer cells in oral squamous cell carcinoma (OSCC) and head and neck squamous cell carcinoma (HNSCC). C3AR expression is associated with cancer stemness markers (CXCL12, NOTCH1) and epithelial–mesenchymal transition (EMT) markers (including SNAIL, VIM, and ZEB1). Adipocytes interact with SCC through C3/C3AR in both autocrine and paracrine fashions, promoting tumor progression. Sphingosine administration inhibits C3/C3AR, leading to the downregulation of C3 expression in OSCC cells [84]. Simultaneously, sphingosine-1-phosphate (S1P), via its receptors S1PR1, activates complement C3 in AKT-dependent manner. Then, active C3 induces cathepsin L (CTSL), a lysosomal protease, that cleaves C3, to generate the active fragments C3b-α′2 and C3a. C3b-α′2 associates with peptidyl-prolyl isomerase-like 1 (PPIL1), which promotes NLRP3 and IL-1β for inflammasome-mediated metastasis. Simultaneously, C3a can bind extracellularly with C3aR and via activation S1P/S1PR1/C3/inflammasome axis mediate lung metastasis [32]. Additionally, complement C3a is highly expressed in the cytoplasm of pancreatic ductal adenocarcinoma (PDAC) cells. C3 promotes the cellular migration and invasion abilities via the Akt and Smad pathways [85]. Yuan et al. demonstrated that intracellular C3 serves as a predictive factor for poor overall survival in gastric cancer. Conclusively, C3 promotes tumor progression by activating the JAK2/STAT3 signaling pathway [86]. C3 is a driving factor of tumor growth by modulation CD8+ T-cells numbers and inducing M2 polarization in tumor-associated macrophages (TAMs). TAMs with enhanced C3 expression exhibit downregulated PI3K/AKT/mTOR signaling. These TAMs produce immunosuppressive cytokines (TGF-β1, TGF-β3, and IL-10) and express immune checkpoint markers of T cell exhaustion (PD-L1, CTLA-4, and TIM3). Importantly, tumor cells with elevated C3 exhibit resistance to the anti-PD-L1 treatment [38].

### 5.4. Extracellular C3/C3aR

In TME of gastric cancer, cancer-associated fibroblasts (CAFs) display elevated C3 expression via the activation of the NF-κB pathway. High C3+CAFs levels correlate with increased EMT activation levels of TNFα and IFN-γ [87]. In renal cell carcinoma (RCC), tumor-derived extracellular vesicles carrying C3 are associated with poor survival. Within the TME of RCC lung metastases, extravesical C3 is absorbed by macrophages, where it initiates the secretion of CCL2 and CXCL1. Upregulated CCL2/CCR2 and CXCL1/CXCR2 pathways are responsible for the recruitment of immunosuppressive TAMs and PMN-MDSCs, leading to a pro-metastatic effect. The whole process is largely C3-dependent, as in an environment without extravesical C3, anti-tumoral immune cell infiltration significantly increases [46]. Similarly, lung mesenchymal stromal cells (LMSCs) in lung metastases exhibit increased C3 expression. The C3–C3a axis promoted neutrophil recruitment into lung tissue. In LMSCs, enhanced C3 expression is driven by STAT6 signaling. Stimulation with IL-13 or IL-4 cytokines, produced by Th2 cells, further amplifies C3 production. Before metastatic colonization, LMSCs are reprogrammed by Th2 cytokines to upregulate C3. This facilitated neutrophil recruitment, promotes the formation of neutrophil extracellular traps (NETs), and thereby traps circulating tumor cells, enabling metastatic colonization [88]. However, C3 can also exhibit tumor-suppressive effects. In triple-negative breast cancer (TNBC), low C3 expression correlates with high levels of cellular apoptosis susceptibility protein (CAS) and with poor prognosis. Silencing of CAS expression leads to an increase in the C3 level, which is associated with reduced tumor growth and metastasis [89].

### 5.5. C3a/C3aR

In multiple myeloma (MM) C3a promotes osteoclast proliferation and differentiation by suppressing Sirt3. C3a increases bone resorption via Sirt1 inhibition and then activation of the PI3K/PDK1/SGK3 signaling pathway [90]. Tumor-associated astrocytes and MB tissues express C3aR with C3a in MB tissue. When co-cultured with astrocytes, MB cells exhibit significantly enhanced tumor cell proliferation. C3a–C3aR engagement triggers the MAPK pathway activation, leading to the elevated secretion of pro-inflammatory cytokines (IL-6 and TNF-α). Whether the source of C3a in MB is extracellular or intracellular is unknown [91]. Additionally, C3a–C3aR promotes tumor metastasis via EMT activation. C3 expression is positively correlated with cancer-associated fibroblast (CAFs) markers and their effector cytokines. CAF infiltration levels show no significant difference between the C3aR+ and C3aR− groups. However, C3aR−/− CAF express significantly lower conventional fibroblast markers (a-SMA, PDGFRα, FAP), altering their function via the PI3K-AKT signaling cascade. It was found that C3aR is a G-protein-coupled receptor, both on the membrane and intracellularly [92].

### 5.6. C3b

C3 insufficiency decreases tumor growth in PTX-resistant small-cell lung cancer. The cleavage product, C3b, modulates tumor cell proliferation via the PI3K/AKT pathway. The C3aR localizes intracellularly rather than on the cell membrane. Only C3b, but not intact C3, is detected intracellularly in the nucleus, and within the cytoplasm of PTX-resistant cancer cells. Nuclear C3b interacts with the HDAC1-containing SIN3A complex and histone-binding proteins RBBP4/7. The C3b–SIN3A complex specifically targets GADD45A, which was suppressed in PTX-resistant tumor cells by inhibiting the histone acetylation of the GADD45D promoter region. The knockdown of SIN3A or HDAC1 sensitizes PTX-resistant tumor cells to PTX treatment [93].

### 5.7. C3d

C3d constitutes a significant factor mediating antitumor activity, overcoming immune evasion. In mice with monoclonal gammopathy, the administration of serum with C3d leads to decreased IgG levels and renders M protein undetectable. Treatment with C3d spares non-malignant cells while targeting most mutated malignant cells. Activity of intracellular C3d alters the expression of Rpl genes, which encode proteins forming the faulty ribosomal subunits in plasma cells. As a result, defective ribosomal products are presented in the enhanced antigen presentation for MHC I. Additionally, C3d activates E2F1-mediated transcription of long non-coding RNAs (lncRNAs) that encode faulty peptides for MHC I binding, further promoting immune recognition [94].

### 5.8. C5a

C5a is significantly expressed on γδ T cells in malignant pleural effusion. C5a effectively stimulates inflammation mediators (IL-17A, IFN-γ, IL-22, CCR2) expression in Vδ2 subtype of γδ T cells. Additionally, C5a promotes the production of chemokines CCL2, CCL7, CCL5, and CCL20 in MPE. As observed, high levels of CCR2 stimulate migration of γδ T into the pleural cavity of MPE patients [95]. In metastatic renal cell carcinoma (mRCC) higher C5a expression correlates with reduced responsiveness to tyrosine kinase inhibitor therapies (sorafenib and sunitinib). C5a stimulation enhances renal cancer cell migration and invasion, accompanied by the increased expression of immune checkpoint proteins PD-L1 and PD-1. Additionally, C5a promotes EMT markers, including the upregulation of vimentin and MMP9 and the downregulation of E-cadherin [96]. Additionally, in gastric (GC) cancer C5a–C5aR1 pathway drives iron accumulation in cancer cells. GC cells induce lipocalin 2 (LCN2) iron transfer from M2 TAMs to cancer cells, promoting GC cells proliferation. Upregulation of LCN2 expression occurs via the activation of endoplasmic reticulum (ER) stress initiated by C5a–C5aR pathway. Additionally, C5a–C5aR1 pathway promotes polarization of TAMs into M2 subtype, which in turn further enhances iron transport [97]. Intracellular C5aR1 is significantly overexpressed in glioblastoma (GBM) and serves as a poor prognostic indicator, promoting the progression of GBM. C5aR1 knockdown decreases glutathione peroxidase 4 (GPX4) levels, promoting lipid peroxidation and ferroptosis of GBM cells. The expression of GPX4 mRNA is inhibited through 6-methyladenosine (m6A) methylation by the methyltransferase METTL3. The expression of METTL3 is upregulated by C5aR1 via the ERK1/2 signaling pathway. Collectively, these findings demonstrate that intracellular C5aR1 inhibits ferroptosis and lipid peroxidation in GBM, through METTL3-dependent m6A methylation of GPX4, thereby enhancing tumor cell survival [98]. Ding et al. observed that the deficiency of C5 and C5a1 prevents artificially induced colorectal cancer (CRC) tumorigenesis. High expression of C3, C5, and C5AR1 significantly correlates with poor overall survival. C5a/C5aR1 signaling also impairs accumulation of CD8+ T cells, MDSCs and reduces number of effector cytokines in the colons. Intense cell proliferation in mice colons only occurs in the presence of C5 and C5aR1, as well as C5a–a product of C5 cleavage at inflamed colorectal sites [99]. High expression of intracellular complement C5a/C5aR1 is also observed in the cytoplasm of human colonic epithelial cells, whereas C5aR1 is present on the surface of neutrophils. C5a and C5aR1 are colocalized in subcellular organelles, including lysosomes and endosomal vesicles. C5aR1 knockdown leads to low levels of β-catenin. However when C5 binds to C5aR1, the C5–C5aR1 stabilizes β-catenin through interaction with the KCTD5 protein, forming a complex with cullin3 and Roc-1 proteins. The C5aR1/KCTD5/cullin3/Roc-1 complex prevents β-catenin degradation by promoting K63-linked polyubiquitination, while inhibiting the K48-linked polyubiquitination. Elevated β-catenin levels increases the transcription of target genes, such as CCND1 and COX2, promoting colorectal tumorigenesis. Inhibition of C5aR1 promotes the destabilization of β-catenin, which subsequently impedes colorectal tumorigenesis by reducing both the number and mass of polyps [28]. In addition, C5a acts as a potent chemoattractant of myeloid-derived suppressor cells (MDSCs), as they have high expression C5aR1. C5a induces the rearrangement of tubulin bundles, enhancing the migration of polymorphonuclear MDSCs (PMN-MDSCs), without affecting monocytic MDSCs. An increase in C5a leads to a reduction in local adhesion clusters and enhances the cell surface expression of CD11b integrin, facilitating endothelium adhesion. Additionally, surface expression of β1 and β3 integrins increases, enabling the proteolytic degradation of collagen. Notably, in the presence of lung carcinoma cells, C5a induces NETosis in PMN-MDSCs via the HMGB1 receptor, promoting lung cancer cell invasion and extracellular matrix degradation. HMGB1 activity is further enhanced by C5a-induced expression of the receptors TLR4 and RAGE in MDSCs [100].

### 5.9. C3 and C5

Ovarian carcinoma (OC) cells co-cultured with adipocytes exhibit increased proliferation associated with C3 and C5 activation from the co-cultured cells. The lipid transport from adipocytes to OC cells is identified as a critical factor of complement protein induction, with the activation of the Src kinase, EGFR, and PI3K/AKT pathways. Additionally, co-culturing adipocytes provokes the stress response, mediated by increased ATF4, contributing to tumor cell survival and growth. Analysis of metastatic tissue from high-BMI patients confirms upregulated C3 expression, supporting its role as a survival factor in OC [101].

### 5.10. Ficolin-3

In cholangiocarcinoma (CCA) cell lines ficolin-3 (FCN3) is significantly downregulated. FCN3 interacts with MASPs from the lectin pathway and enhances the formation of complement component C3b and C5b-9, promoted complement-dependent cytotoxicity with elevated levels of necroptosis-associated proteins. FCN3-overexpressing CCA cells exhibit necroptosis features, including membrane rupture, organelle swelling, disintegration of the nucleus and cytoplasm, and increased numbers of necrotic and apoptotic cells [102].

### 5.11. Factor H

Complement factor H (FH) acts as a ligand Inducible T-cell Costimulator (ICOS) within the glioma microenvironment. The FH–ICOS signaling induces Akt pathway phosphorylation and glycogen synthase kinase-3. FH binds to ICOS on both CD4+ and CD8+ T cells, but increases the viability only of Tregs and CD4+ T cells. Additionally, activation of ICOS induces stimulation of immunosuppressive cytokines, IL-10 secretion and TGFβ. In glioma microenvironment, FH may affect various immune checkpoints–promoting CTLA-4 expression and PD-L1 upregulation, while interfering with PD-1 [103]. Intracellular factor H stimulates proliferation and motility of clear cell renal cell carcinoma (ccRCC) and lung adenocarcinoma (ADC) cells. FH is mainly localized in lysosomes and on cell membrane. The silencing of FH inhibits cell proliferation and motility through p53 phosphorylation and NF-κB nuclear translocation [104].

### 5.12. CD46

In bladder cancer cells, CD46 induces the upregulation of KRT13, GABRP, and C3-α, significantly enhancing motility and migration [105]. CD46 enhances bladder cancer cells’ resistance to the inhibitory effect of cetuximab (an EGFR inhibitor) by modulating AKT and ERK phosphorylation. Additionally, CD46 protects from complement-mediated lysis and antibody cell-mediated cytotoxicity [106]. In a recent study, CD46 induced expression of the transcription factor AP-1, activating the p38 MAPK and AKT pathways. Then, via AP-1–dependent c-Jun phosphorylation, MMP9 synthesis and activity are enhanced, further promoting the migration and invasion of cancer cells [107].

### 5.13. CD55, CD59

CD55 is responsible for the induction of cisplatin resistance and stemness in ovarian cancer (OC) cells through a novel mechanism within the nucleus. When glycosylated at the cell surface, CD55 migrates to the nucleus. The migration to the nucleus is dependent on the presence of serine/threonine (S/T) domains near the membrane, which enable chromatin binding. Within the nucleus of OC cells, CD55 disrupts the suppressor complex ZMYND8 with EZH2 and SUZ12. This results in increased histone H3 trimethylation, silencing tumor suppressor genes [108]. In lung cancer CD55 and CD59 expression is enhanced by activation of epidermal growth factor. The upregulation of CD55/CD59 significantly reduces the cytotoxic activity of CD8+ T cells, decreasing levels of effector molecules granzyme B, perforin, IFN-γ, TNF-α, and various interleukins (IL-6, IL-1β, IL-17). CD55/CD59 expression is enhanced by suppression of CD55-targeting miR-216b and CD59-targeting miR-150 via upregulation of β-catenin-mediated LINC00973- lncRNA with binding sites for both miR-216b/miR-150. Beyond tumor evasion, the EGFR/Wnt/β-catenin-upregulated CD55/CD59 cascade promotes resistance to anti-PD-1 therapy [109]. Additionally, CD59 expression in immune cells is crucial for tumor growth. The CD59 abundance on the membrane is significantly lower compared to total expression in colorectal cancer and lung cancer, thus acting complement independently. CD59 interacts with the Ras protein, facilitating its transport to the plasma membrane of T cells. In contrast, CD59 deletion results in Ras being retained within the Golgi apparatus. When Ras is trapped in the Golgi, there is increased activation of MAPK molecules, leading to enhanced T-cell proliferation and increased cytotoxic function [110].

### 5.14. Factor I

Factor I presence in non-small-cell lung cancer (NSCLC) has a negative impact on survival. In cancer tissue, there is no correlation between FI and its target C4d complement, suggesting that FI may act in a non-canonical way. In the cells expressing FI, there is observed significant phosphorylation of JNK/p38 MAPK pathway and IRS-1, GSK3 signaling. Moreover, FI-sufficient cells exhibits significant resistance to programmed cell death via etoposide and they create larger colonies than FI-deficient ones [111].

### 5.15. Factor B

Intrinsic and extrinsic complement factor B (CFB) expression is observed in cells of pancreatic ductal adenocarcinoma (PDAC). CFB knockdown does not affect EMT markers (E-cadherin, vimentin), nor it affects apoptotic cell death. However, CFB depletion decreases cell proliferation, by repressing the S phase of cell cycle. Moreover, the silencing of CFB expression increases number of senescence-associated-β-galactosidase (SA-β-gal) cells with increased p21 and cyclin D1 expression, showing the induction of the senescence in PDAC cells. Additionally, high CFB expression induced high percentage of Foxp3+ Tregs, further underscoring immunosuppressive role of CFB in antitumor response [112].

**Table 3:** Roles of extracellular complement components in cancer by localization.

Component	Cancer Types	Key Mechanisms	Effects	Pathways
C1q/C1qR [78]	MPM	HA degradation into LMW-HA via HYAL2	Pro-inflammatory, pro-tumorigenic effect on TME	Not determined
C1r/C1s [80,81,83]	cSCC, RCC	MMP1, MMP13, MMP10, and MMP12 upregulation; collagen degradation,	Invasion of tumor cells, proliferation, negative prognosis in early stages of RCC	Akt, ERK1/2
C3/C3aR [51,87,88,89]	OSCC, cSCC, RCC, Lung metastasis, TNBC	CAF/PMN-MDSC recruitment; senescence; upregulation of pro-inflammatory mediators; increase in CAS in TNBC; EMT activation	Immunosuppression, metastasis, induction of apoptosis in TNBC, proliferation	NF-κB, STAT6
C3a [90,91,92]	Medulloblastoma, MM, breast cancer	CAF EMT; Sirt1 suppression; cytokines astrocyte/MAPK activation	Metastasis, proliferation, osteoclastogenesis	PI3K/AKT PI3K/PDK1/SGK3, MAPK
C3d [94]	Monoclonal gammopathy	MHC I via faulty ribosomes/lncRNAs	Anti-tumor immunity	E2F1
C5a/C5aR [95,96,97,99,100]	mRCC, GC, CRC, lung cancer	PD-L1/EMT; TLR4 and RAGE receptors induction; γδ T-cell chemoattraction, β-catenin stabilization; LCN2-mediated iron transfer	M2 TAMs promotion, tumor evasion, increased proliferation and invasion	ERK1/2
C3/C5 [101]	Ovarian cancer	Adipocyte lipid-induced activation; increased ATF4 level	Proliferation, ISR survival	Src kinase, EGFR, PI3K/AKT
Ficolin-3 [102]	Cholangiocarcinoma	MASP/C3b/C5b-9; necroptosis induction	Anti-tumor lysis promotion	RIPK/MLKL
Factor H [103]	Glioma	ICOS signaling; cytokine secretion; CTLA-4 and PD-L1 upregulation; PD-1 inhibition	Treg population expansion, tumor evasion	Akt/GSK3
CD46 [105,106,107]	Bladder, Gallbladder cancer	KRT13, GABRP, and C3-α upregulation; transcription factor AP-1 induction; increased c-Jun phosphorylation; increased MMP9 secretion; EGFR resistance	Tumor invasion, lysis protection;	p38 MAPK, AKT
CD55/CD59 [109]	NSCLC	EGFR/Wnt/β-catenin-upregulated CD55/CD59 cascade	T-cell/macrophage complement mediated cytotoxicity inhibition, tumor evasion, resistance to anti-PD-1 inhibitors	EGFR/Wnt/β-catenin
Factor B [112]	PDAC	Stromal senescence block	Treg cells, PMN-MDSCs, TAMs induction, poor prognosis, increased proliferation	Not determined

Abbreviations. Cancer Types: CRC: Colorectal Cancer; cSCC: Cutaneous Squamous Cell Carcinoma; GC: Gastric Cancer; MPM: Malignant Pleural Mesothelioma; MM: Multiple Myeloma; mRCC: Metastatic Renal Cell Carcinoma; NSCLC: Non-Small Cell Lung Cancer; OSCC: Oral Squamous Cell Carcinoma; PDAC: Pancreatic Ductal Adenocarcinoma; RCC: Renal Cell Carcinoma; TNBC: Triple-Negative Breast Cancer. Key Mechanisms: Akt: Protein kinase B; AP-1: Activator protein 1; β-catenin: Beta-catenin; C3-α: alpha chain of complement factor C3; CAF: Cancer-Associated Fibroblasts; CAS: Chromosomal instability; EGFR: Epidermal Growth Factor Receptor; EMT: Epithelial–Mesenchymal Transition; ERK: Extracellular signal-regulated kinase; E2F1: E2F transcription factor 1; GABRP: GABA A receptor pi subunitGSK3; Glycogen Synthase Kinase 3; HA: Hyaluronic Acid; HYAL2: Hyaluronidase 2; ICOS: Inducible T-cell COStimulator; ISR: Integrated Stress Response; KRT13: Keratin 13; LMW-HA: Low Molecular Weight Hyaluronic Acid; MAPK: Mitogen-Activated Protein Kinase; MASP: MBL-associated Serine Protease; MDSC: Myeloid-Derived Suppressor Cells; MMPs: Matrix Metalloproteinases; NF-κB: Nuclear Factor kappa-light-chain-enhancer of activated B cells; PD-L1: Programmed Death-Ligand 1; PDK1: 3-phosphoinositide-dependent kinase-1; PI3K: Phosphoinositide 3-Kinase; PMN-MDSC: Polymorphonuclear Myeloid-Derived Suppressor Cells; RIPK: Receptor-Interacting Protein Kinase; SGK3: Serum/Glucocorticoid Regulated Kinase 3; Src: Proto-oncogene tyrosine-protein kinase Src; STAT6: Signal Transducer and Activator of Transcription 6; TAM: Tumor-Associated Macrophage; TME: Tumor Microenvironment; Tregs: Regulatory T Cells; Wnt: Wingless/Integrase-1 signaling pathway.

**Table 4:** Roles of complosome components in cancer by localization.

Component	Cancer Types	Key Mechanisms	Effects	Pathways
C1q/C1qR [79]	Colorectal	Caspase-1 cleavage of C1qR blocks mitochondrial import	Increase in aerobic glycolysis and proliferation	Not determined
C1r/C1s [82]	cSCC	Stimulation of tumor vascularization and tumor cell viability; inhibition of apoptosis and MMP-9 production	Proliferation/invasion	Akt, ERK1/2
C3/C3aR [32,38,84,85,86]	OSCC, HNSCC, PDAC, Gastric	Stemness/EMT induction; CTSL/NLRP3 activation and C3 enzymatic cleavage;	Migration, inflammasome-mediated metastasis, M2 polarization, tumor evasion, resistance to anti-PD-1 therapy	AKT, JAK2/STAT3, PI3K/AKT/mTOR, S1P/S1PR1/C3/inflammasome, Smad
C3b [93]	SCLC	Nuclear SIN3A/HDAC1 suppressing GADD45A	PTX resistance; proliferation	PI3K-AKT
C3a [92]	Breast cancer	CAF EMT	Metastasis	PI3K-AKT
C5aR1 [28,98]	GBM, CRC	GPX4 m6A; β-catenin stabilization (KCTD5/cullin3)	Ferroptosis inhibition; lipid peroxidation; tumorigenesis; increased tumor cell survival	ERK1/2, METTL3
Factor H [104]	ccRCC, Lung ADC	Not determined	Motility, morphology, poor prognosis,	p53, NF-κB
CD55 [108]	Ovarian cancer	Nuclear ZMYND8/EZH2 disruption	Cisplatin resistance, cancer cell stemness, cell proliferation promotion	PRC2/H3K27
Factor I [111]	NSCLC	IRS-1 phosphorylation, GSK3β phosphorylation	Apoptosis resistance, poor prognosis,	JNK/p38/IRS-1, JNK/p38/GSK3
CD59 [110]	Lung cancer	Intracellular Ras retention in Golgi	Increased T-cell activation, anti-tumor response	Ras/MAPK

Abbreviations. Cancer Types: ADC: Adenocarcinoma; ccRCC: Clear Cell Renal Cell Carcinoma; CRC: Colorectal Cancer; cSCC: Cutaneous Squamous Cell Carcinoma; GBM: Glioblastoma Multiforme; HNSCC: Head and Neck Squamous Cell Carcinoma; NSCLC: Non-Small Cell Lung Cancer; OSCC: Oral Squamous Cell Carcinoma; PDAC: Pancreatic Ductal Adenocarcinoma; SCLC: Small Cell Lung Cancer. Key Mechanisms: AKT: Protein kinase B; β-catenin: Beta-Catenin; CAF: Cancer-Associated Fibroblasts; CTSL: Cathepsin L; EMT: Epithelial–Mesenchymal Transition; ERK1/2: Extracellular Signal-Regulated Kinases 1 and 2; EZH2: Enhancer of Zeste Homolog 2; GADD45A: Growth Arrest and DNA Damage-Inducible 45 Alpha; GPX4: Glutathione Peroxidase 4; GSK3β: Glycogen Synthase Kinase 3 Beta; HDAC1: Histone Deacetylase 1; IRS-1: Insulin Receptor Substrate 1; JAK2: Janus Kinase 2; JNK: c-Jun N-terminal Kinase; KCTD5: Potassium Channel Tetramerization Domain-Containing 5; MAPK: Mitogen-Activated Protein Kinase; METTL3: Methyltransferase-like 3; MMP-9: Matrix Metalloproteinase 9; m6A: N6-methyladenosine; NLRP3: NOD-, LRR-, and pyrin domain-containing protein 3; NF-κB: Nuclear Factor kappa-light-chain-enhancer of activated B cells; PD-1: Programmed Cell Death Protein 1; PI3K: Phosphoinositide 3-Kinase; PRC2: Polycomb Repressive Complex 2; PTX: Paclitaxel; Ras: Rat Sarcoma Proto-Oncogene; S1P: Sphingosine-1-Phosphate; S1PR1: Sphingosine-1-Phosphate Receptor; SIN3A: SIN3 transcription regulator family member A; STAT3: Signal Transducer and Activator of Transcription 3; ZMYND8: Zinc Finger MYND-Type Containing 8.

## 6. Conclusions

The growing recognition of the complosome as a regulator of intracellular signaling in human diseases has stimulated the development of therapeutic strategies targeting the complement system at defined anatomical sites [77]. For example, in age-related macular degeneration (AMD), complement inhibitors are administered via intravitreal injection [113], whereas in periodontal diseases, local delivery into the oral cavity is successfully explored [29]. In parallel, advanced approaches such as adeno-associated virus (AAV)-mediated gene delivery [114] and bispecific molecules are being developed to modulate complement activity with improved specificity [115].

However, a major and still unresolved challenge is the intracellular localization of the complosome, which limits the effectiveness of conventional extracellular complement inhibitors and necessitates the development of cell-permeable or intracellularly targeted therapeutics [77].

Despite rapid progress, several critical knowledge gaps remain. First, the cell-type-specific functions of the complosome across tumor cells, immune cells, and stromal populations are not yet fully defined, particularly in the context of dynamic tumor microenvironments. Second, there is a lack of effective strategies for the selective delivery of intracellular complement inhibitors, especially in vivo, where issues of targeting, stability, and off-target effects remain unresolved. Third, the potential of complosome components as diagnostic and prognostic biomarkers in cancer and other diseases remains underexplored and requires systematic clinical validation.

Addressing these challenges will be essential to fully understand the role of the complosome as a regulator of cellular metabolism, immune function, and stress adaptation, and to translate this knowledge into effective therapeutic interventions. Overall, targeting the intracellular complement system represents a promising but still emerging direction in precision medicine.its therapeutic potential in autoimmune, infectious, and cancerous diseases.

## Figures and Tables

**Table 1:** Complosome Effects on Immune Interactions in Tumors.

Immune Cell Type	Complosome Driven Mechanism	Functional Outcome
CD4+/CD8+ T cells [32,42,43,44]	Intracellular C3/C5 signaling alters metabolism; C5aR-driven suppression; MDSC-mediated inhibition	T-cell exhaustion, reduced cytotoxicity, increased Tregs
Macrophages (TAMs) [41,45,46,47]	CFI suppresses intracellular C5a; C1q/iC3b skewing; classical pathway activation with tumor cells	M2 polarization, immune checkpoint upregulation, enhanced tumor support
MDSCs [44]	C5a enhances ROS/RNS production, increases suppressive capacity	Strong inhibition of T-cell function
Diverse immune cells via metabolic control [27,32]	Complosome regulates glycolysis, OXPHOS, mitochondrial homeostasis	Reduced immune effector function in TME; metabolic exhaustion
CAR-T cells (engineered) [48]	Increased intracellular complement activation enhances DNA repair and lowers exhaustion	Improved antitumor immunity

**Table 2:** Immune Cell Responses to Complosome Signals in Cancer.

Immune Cell Type	Key Response to Complosome Signals
CD4+/CD8+ T cells [35,51,59,60]	Metabolic rewiring via intracellular C3; suppression/exhaustion via C5a–C5aR1; reduced infiltration from EV-C3 environments
Macrophages (TAMs) [50,51,61]	M2 polarization via C3/C3a/C3aR and C5aR1; secretion of immunosuppressive cytokines; recruitment of suppressor cells
MDSCs [51,62]	Enhanced recruitment and suppressive activity driven by C5a
Diverse immune cells [27,59,63]	Reprogrammed immunometabolism from intracellular complement activity

## Data Availability

No new data were created or analyzed in this study. Data sharing is not applicable to this article.
